# Leigh's Disease: The Acute Clinical Course of a Two-Year-Old Child with Subacute Necrotizing Encephalomyelopathy

**DOI:** 10.1155/2010/986302

**Published:** 2010-06-10

**Authors:** Bettina Zinka, Andreas Buettner, Matthias Graw

**Affiliations:** Institute of Legal Medicine, University of Munich, Nussbaumstraße 26, 80336 Munich, Germany

## Abstract

We report the untypical clinical course of a previously healthy two-year-old girl, who died suddenly and unexpectedly after an episode of vomiting. At forensic autopsy no other pathological findings could be diagnosed than multiple reddish, sunken areas in brain stem, mesencephalon, and pons. Histologically they presented as areas of spongiosis of the neuropil with prominent endothelial hyperplasia and vascular proliferation whereas nerve cells were well preserved. On the basis of the characteristic neuropathological findings in combination with the age of the child, we had to take into consideration that the child might have died from subacute necrotizing encephalomyelopathy (Leigh's Disease) despite the untypical, fulminant clinical course.

## 1. Introduction

Normally subacute necrotizing encephalomyelopathy presents with a clinical course of at least several months and impressive clinical signs. But possibly there are undocumented, unexpected deaths which could have been diagnosed as subacute necrotizing encephalopathy only in postmortem examiniation, if performed, due to atypical progression.

## 2. Case Report

A previously healthy 24-month-old girl with strabismus concomitans, associated with a nystagmus, presented to a pediatric hospital. Furthermore the girl's mother reported unspecific symptoms like attention deficits, decrease in alertness, increased nervousness and “interaction problems”. 

On examination the girl was found to be in general good health with appropriate nutrition. Physical and psychomotor development was documented to be normal. Clinical examinations, including laboratory examinations as well as a neurological examination with magnetic resonance imaging (MRI), which was performed as a precaution to exclude an intracranial disease, were all found to be normal. Electroencephalography showed a small decrease in alertness associated with sharp-wave and theta rhythm activity. Ophthalmologically a strabismus concomitans divergence was diagnosed. The child was allowed to return home but ophthalmologic follow-up was recommended.

Three months later the child started vomiting at home. Finally the paramedics were called as the girl lost hemorrhagic mucus from her nose. Shortly afterwards she lost consciousness and when the paramedics arrived she was already clinically dead. Reanimation was unsuccessful. Death was attributed to aspiration during an assumed epileptic seizure.

Forensic postmortem examination of the girl showed few irregularities; in particular the heart and lungs were free of any pathological findings. Laboratory examinations of glucose metabolism as well as chemical-toxicological studies showed normal results except for increased lactate concentration in blood (47.9 mmol/l; refernce: <2.4 mmol/l) and cerebrospinal fluid (26.3 mmol/l; reference: 1.1–2.1 mmol/l). 

**Figure 1:**
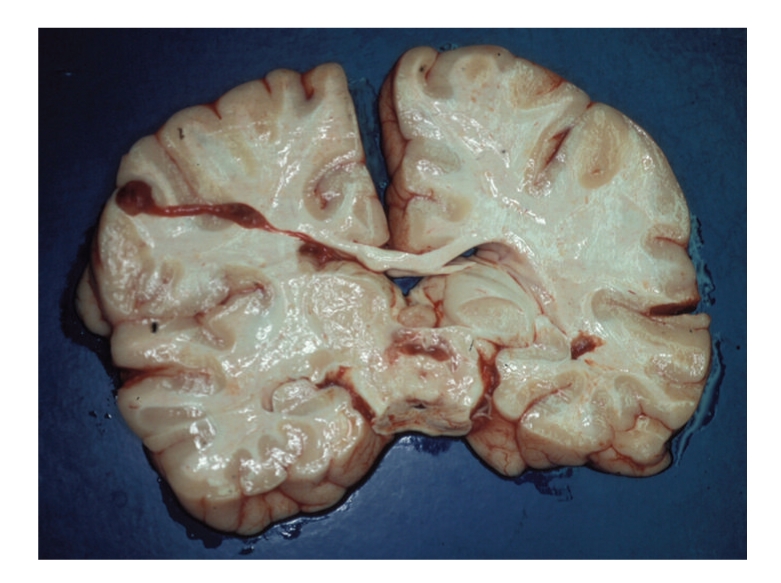
Reddish, sunken foci within the pons.

**Figure 2:**
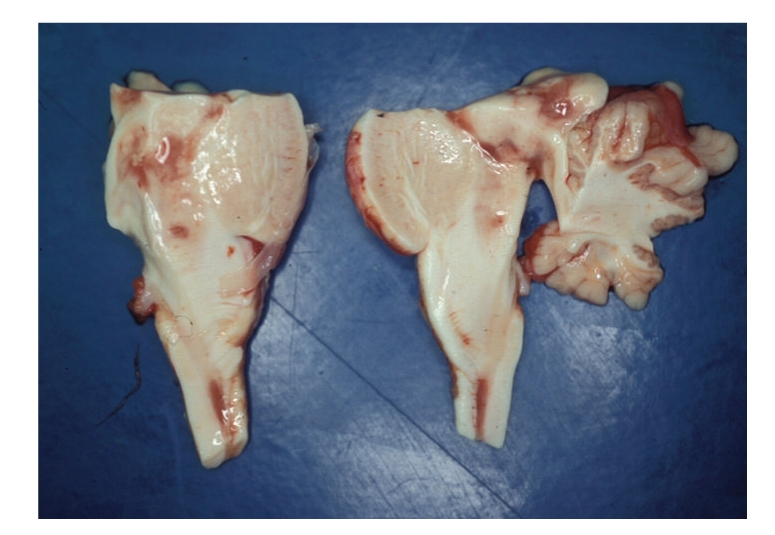
Cystic, reddish areas in mesencephalon, pons, and medulla oblongata.

The brain weighted 1061 g. Leptomeninges and gyration patterns were unremarkable; marked signs of edema and significant tonsillar herniation were seen. However, on horizontal sections small reddish foci of tissue softening with cystic appearance were seen within the pons, mesencephalon, and medulla oblongata (Figures 1 and 2).

After fixation the brain was histologically examined (hematoxylin and eosin, cresyl-violet, Luxol-Fast-Blue, van-Gieson Elastica, PAS, and Prussian Blue). 

Deparaffinized 5-*μ*m-thick sections were immunostained with the ABC method. For labelling astrocytes a monoclonal antibody against glial fibrillary acidic protein (GFAP, dilution 1:100, DAKO, Germany) was used; for microglia a monoclonal antibody against the HLA-DP, DQ, DR antigen (CR 3/43, dilution 1:100, DAKO, Germany, predigestion with formic acid) was used. Antigens were detected with a Histostain-Plus Peroxidase Kit (Zytomed, Germany).

**Figure 3:**
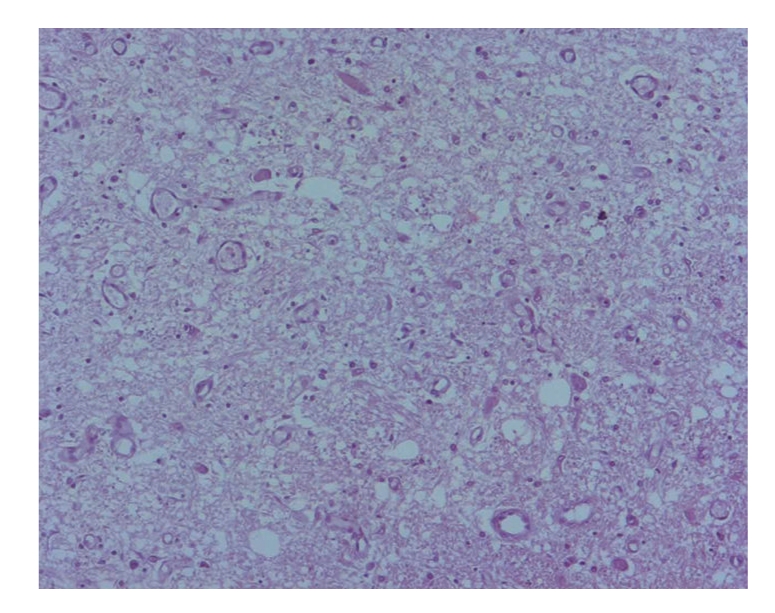
Histological picture of the macroscopic lesion of the pons (HE, 100-fold).

On histological examination there was widespread hypoxic-ischemic nerve cell damage within the major lobes, dentate nucleus, and Purkinje cell layer. On sections of the caudate nuclei, thalamus, hypothalamus, mesencephalon, pons, and medulla oblongata there were widespread foci of spongiosis of the neuropil with prominent endothelial hyperplasia and vascular proliferation. Besides hypoxic-ischemic changes, the nerve cells were relatively well preserved (Figure 3). Immunostaining for GFAP revealed marked astrogliosis. The white matter of the cerebral hemispheres, the hippocampal formation, the medulla oblongata, and the mamillary bodies were unremarkable.

## 3. Discussion

The rare subacute necrotizing encephalomyelopathy was first described by Leigh [1] as a clinically and etiologically highly variable, progressive syndrome seen particularly in pediatric population although a few juvenile and adult cases are known [2, 3].

The unspecific, heterogenic, and apparently age-dependent variation in clinical symptoms and course may possibly be attributed to identification of different enzyme defects in this disease. Already mitochondrial as well as nuclear DNA enzyme defects have been found [2, 4, 5] that determine similar clinical courses but occur at different ages. Clinical symptoms are also determined by which area of the brain is affected. 

The infantile form usually begins during the first two years of life with unspecific symptoms and a slowing in psychomotor development. Other symptoms include attention deficit, limb hypotonia, visual irregularities including nystagmus, poor strength, emesis, unwillingness to eat, and loss of weight [2, 4, 6]. Neurological symptoms such as ataxia, deficits of the pyramidal tract, apraxia, and myoclonus often develop, followed by ophthalmoplegia and respiratory difficulties as a sign of brain stem damage [2].

Congenital cytochrome-c-oxidase deficiency, pyruvate-dehydrogenase deficiency as well as biotinidase deficiency are known to cause these clinical symptoms [4, 7, 8]. These states probably have an autosomal-recessive inheritance although mitochondrial and maternal inheritance are being discussed and sporadic cases are known [3, 4]. Genetic irregularities could only be found in approximately 50% of all patients, showing that there are many other biochemical irregularities which could lead to the clinical syndrome of Leigh's disease [8].

Specific biochemical tests are still lacking, however lactate acidosis and increased lactate-pyruvate quotient in plasma and CSF are nearly always found [4]. The search for mitochondrial DNA mutations as a common cause for Leigh's Disease and biochemical investigation of muscle biopsies still remain the gold standard in diagnosis [9, 10]. 

On neuropathological examination of the brain symmetrical, central lesions of red brown colour in areas of the brain stem, putamen, basal ganglia, thalamus, and posterior horn of the spinal cord can macroscopically be identified [6].

In early stage neuropil is damaged by spongiosis, followed by capillary proliferation, astrogliosis, and increased numbers of macrophages. In the course of the disease demyelination occurs, which leaves neurons and axons nearly intact. That allows, for example, differentiation from ischemic/hypoxic lesions [1, 2, 6, 7]. In particular the mesencephalon, pons, caudate, medulla oblongata, central cranial nerve nuclei, inferior olivary nuclei, cerebellum, basal ganglia, and the optic nerve are affected [6].

Destruction of cerebral tissue causes progressive neurological dysfunction, which regularly ends in death at about two years after diagnosis due to central respiratory paralysis. Currently there exists no curative therapy [3, 4, 6]. 

MRI shows symmetrical, hypodense (T1) and hyperdense (T2) lesions in the basal ganglia, involving especially the area of the caudate and putamen. Similar lesions are also seen in Wernicke's encephalopathy, multiple sclerosis, Wilson's disease, amino acid urea, mitochondrial encephalomyelopathies, and various forms of intoxication [3]. In contrast to Wernicke's encephalopathy mamillary bodies are spared from pathological changes [3]. A differentiation to Wilson's disease is possible through laboratory studies of the blood [4]. 

At the moment most symptomatic therapies try to improve the oxidative and bioenergetic capabilities of the mitochondria [6]. The use of coenzyme Q10, thiamine, and carnitine may cause a decrease in severity of symptoms in a few cases [6]. Cacic et al. report that a 19-year-old man symptoms were kept stable for at least four years by a therapy including levodopa, carbidopa, and creatinine monohydrate [8].

## 4. Conclusions

In the presented case the diagnosis of Leigh's Disease could only be reached postmortem although three months prior to death MRI scans and laboratory examinations had been performed. They showed normal findings, furthermore other typical symptoms—like delay in psychomotor development, weakness, eating difficulty, or typical neurological symptoms—were not observed. The clinical symptoms on the day of death were probably primarily of acute cerebral origin. However, postmortem examination showed morphological findings of the child's brain which are specific to Leigh's disease. An increased lactate concentration in blood and CSF could be confirmed in postmortem examination, which is typical to Leigh's disease, but can be seen in many autopsies because of the generalized agonal cellular hypoxemia. 

It is to be assumed that this case report describes an unusual, “acute” course of the normally subacute necrotizing encephalomyelopathy with a strikingly short and mostly unnoticeable clinical course.
